# Primary Sequence and 3D Structure Prediction of the Plant Toxin Stenodactylin

**DOI:** 10.3390/toxins12090538

**Published:** 2020-08-21

**Authors:** Rosario Iglesias, Letizia Polito, Massimo Bortolotti, Manuela Pedrazzi, Lucía Citores, José M. Ferreras, Andrea Bolognesi

**Affiliations:** 1 Department of Biochemistry and Molecular Biology and Physiology, Faculty of Sciences, University of Valladolid, E−47011 Valladolid, Spain; riglesia@bio.uva.es (R.I.); luciac@bio.uva.es (L.C.); 2 Department of Experimental, Diagnostic and Specialty Medicine—DIMES, General Pathology Section, Alma Mater Studiorum—University of Bologna, Via S. Giacomo 14, 40126 Bologna, Italy; letizia.polito@unibo.it (L.P.); massimo.bortolotti2@unibo.it (M.B.); manuela_pedrazzi@hotmail.com (M.P.)

**Keywords:** 3D structure, plant toxin, primary sequence, ribosome-inactivating protein, stenodactylin, toxic lectin

## Abstract

Stenodactylin is one of the most potent type 2 ribosome-inactivating proteins (RIPs); its high toxicity has been demonstrated in several models both in vitro and in vivo. Due to its peculiarities, stenodactylin could have several medical and biotechnological applications in neuroscience and cancer treatment. In this work, we report the complete amino acid sequence of stenodactylin and 3D structure prediction. The comparison between the primary sequence of stenodactylin and other RIPs allowed us to identify homologies/differences and the amino acids involved in RIP toxic activity. Stenodactylin RNA was isolated from plant caudex, reverse transcribed through PCR and the cDNA was amplificated and cloned into a plasmid vector and further analyzed by sequencing. Nucleotide sequence analysis showed that stenodactylin A and B chains contain 251 and 258 amino acids, respectively. The key amino acids of the active site described for ricin and most other RIPs are also conserved in the stenodactylin A chain. Stenodactylin amino acid sequence shows a high identity degree with volkensin (81.7% for A chain, 90.3% for B chain), whilst when compared with other type 2 RIPs the identity degree ranges from 27.7 to 33.0% for the A chain and from 42.1 to 47.7% for the B chain.

## 1. Introduction

Ribosome-inactivating proteins (RIPs) are a family of enzymes widely spread throughout the plant kingdom. RIPs are found in different angiosperms and are also present in some fungal and bacterial species [1]. Many RIP-producing plants have been used for centuries in traditional medicine, and they are still used in folk medicine against several pathologies [2,3]. RIPs possess rRNA N-glycosilase and polynucleotide: adenosine glycosilase activities; RIPs are able to remove one or more adenine from rRNA and several other polynucleotide substrates, thus causing ribosome damage and cell death [1,4,5]. Based on their structure, RIPs are divided into two main groups: type 1 and type 2. The first group consists of RIPs characterized by a single polypeptide chain, of about 30 kDa, with enzymatic activity. The second group includes toxins, with molecular weight of 60–65 kDa, consisting of two polypeptide chains: an enzymatically active A-chain, with properties similar to type 1 RIPs, linked through a disulfide bond to a B-chain with lectin properties. The B-chain has strong affinity for sugar moieties on the cell surface and can facilitate the entry of the toxin into the cell, thus conferring to many type 2 RIPs high cytotoxic effect [6].

Amongst type 2 RIPs, one of the most potent is stenodactylin, purified from the caudex of *Adenia stenodactyla* Harms, a tropical plant belonging to the Passifloraceae family. This RIP has a high enzymatic activity toward ribosomes and herring sperm DNA substrates and is specific for galactose [7]. Interestingly, stenodactylin showed a very low median lethal dose for mice, 2.76 µg/kg at 48 h [7], comparable or lower than the LD_50_s reported in the literature for ricin, ranging from 2 to 22 µg/kg [6].

Like other RIPs purified from *Adenia* species, namely, modeccin and volkensin [8], stenodactylin is retrogradely transported when injected into the central nervous system [9]. This property could have several medical and biotechnological applications in the field of neuroscience to selectively lesion specific neurons.

It has been reported that in a neuroblastoma cell line, stenodactylin can induce multiple cell death pathways, involving mainly apoptosis, but also necroptosis and the production of free radicals [10]. Similar results have been recently obtained in acute myeloid leukemia cells, in which stenodactylin can elicit a rapid stress response with production of pro-inflammatory factors and oxidative stress leading mainly to apoptosis, but also triggering other cell death pathways [11].

RIPs have been studied for many years because of their therapeutic use as toxic moieties of immunotoxins, chimeric molecules obtained conjugating a cytotoxic RIP to a specific carrier, mainly a monoclonal antibody, thus allowing for the selective killing of target cells. Immunotoxins have been included in several clinical trials against various diseases, often achieving promising results, especially in the treatment of hematological neoplasms [12,13]. Due to its high cytotoxic potential, stenodactylin could represent an ideal candidate both as toxic moiety of immunotoxins for the treatment of cancers, and as a single agent for loco-regional treatments. In order to envisage such uses, the knowledge of the primary sequence of stenodactylin and the comparison with the amino acid sequence of other RIPs are essential, thus providing useful information about cell interaction and the toxicity mechanism of stenodactylin.

Through comparing amino acid sequences of RIPs, a high similarity can be observed between type 1 and the A chains of type 2 RIPs and among the B chains of type 2 RIPs. However, the primary structure homologies can vary from 15 to 80% between RIPs from different species [14]. X-ray diffraction analyses showed that the 3D-structures of RIPs are well conserved, with differences in the only C-terminal region and the surface loop structure. Ricin was the first RIP to be analyzed by X-ray diffraction. Ricin A chain is a globular protein that is folded into three domains that largely exhibit α-helical and β-strand structures [15]. The A chain includes two N-glycosylation sites (Asn10-Phe11-Thr12 and Asn236-Gly237-Ser238), but these sites do not appear to be important for proper folding [16]. Ricin B chain consists of two topologically similar domains (lectins), composed by four subdomains (1λ, 1α, 1β and 1γ for domain 1 and 2λ, 2α, 2β and 2γ for domain 2). Only 1α and 2γ subdomains demonstrated galactose-binding activity through a network of hydrogen bonds [15].

Crystallization and preliminary X-ray diffraction data analyses of stenodactylin have already been reported [17], but very little is known about the protein sequence level. Only the first 22 and 21 amino acid residues of the A and B chains have been determined using direct Edman degradation. A protein sequence alignment between stenodactylin, modeccin (*A. digitata*), lanceolin A1, lanceolin A2 (*A. lanceolata*) and volkensin (*A. volkensii*) showed that the A chain of stenodactylin shares 21/21 identity with lanceolin A2 and 15/21 with volkensin. The identity among the B chains is also very high, except for the first three N-terminal residues; the sequence Asp-Pro-Valis present only in the stenodactylin and volkensin B chains [7].

In this work we report the complete amino acid sequence of stenodactylin. The comparison of the stenodactylin primary sequence with that of the other RIPs and the homology degree allowed us to identify the amino acids directly or indirectly involved in RIP toxicity.

## 2. Results

### 2.1. Stenodactylin Gene Sequence

**Figure 1:**
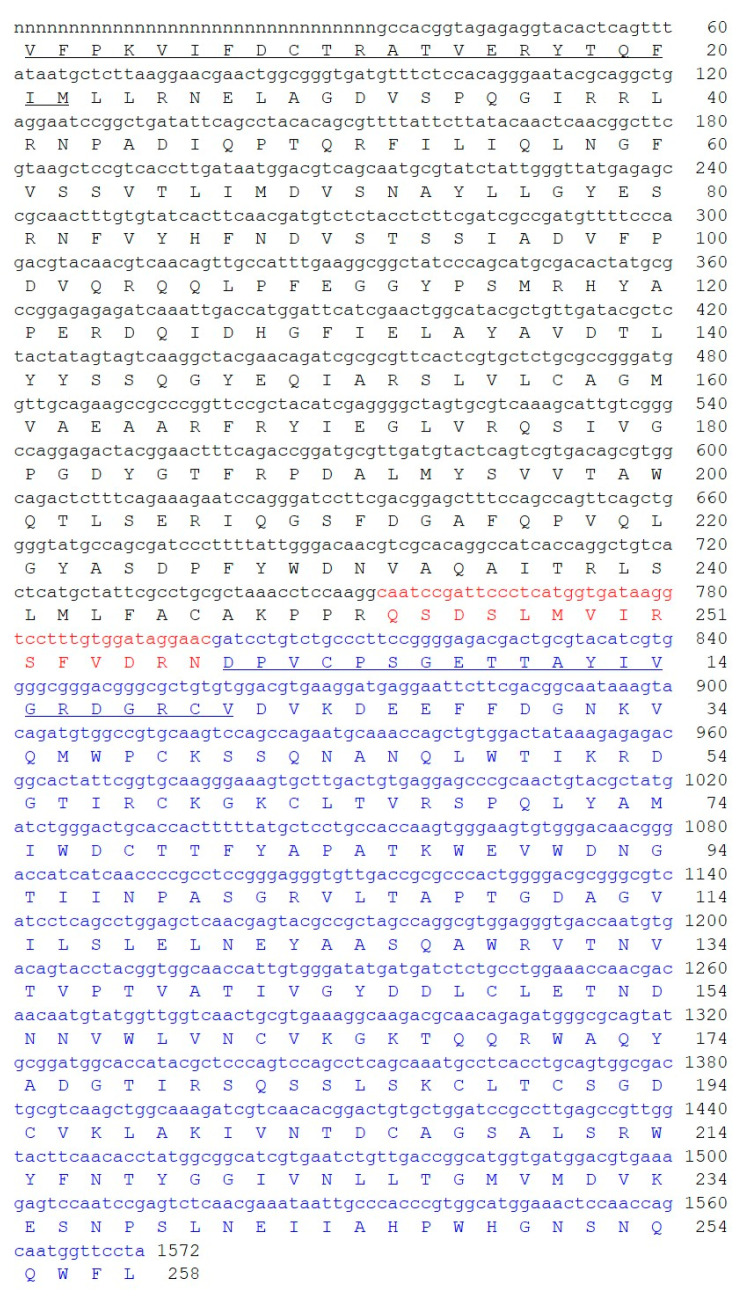
Full length sequence and derived amino acid sequence of the stenodactylin gene. The A chain is presented in black; the B chain is presented in blue and the sequence of the connecting peptides is presented in red. The amino acid sequences obtained by Edman degradation, as described in [7], are underlined. Numbering refers to the position of the amino acids in the mature A and B chains. The cDNA sequence for stenodactylin was submitted to GenBank (accession number: MT580807). The letter “n” means “unknown nucleotide residue”, being the amino acid sequence obtained exclusively by Edman degradation.

Based on of the N-terminal amino acid sequences of the A and B chains of stenodactylin previously obtained by Edman degradation [7] and based on the amino acid sequence of volkensin (CAD61022), five specific primers were designed for the PCR amplification of stenodactylin cDNA (see Section 4.2.1). Three primer pairs were used to amplify the A chain (STA2-STB1R), the A chain with part of the B chain (STA2-STB3R) and the B chain (STB1-STB5R) of stenodactylin. The fragments corresponding to the stenodactylin gene ends presented a homologous overlapping region. The sequence information was analyzed using the algorithms available at http://expasy.org [18]. Excluding the nucleotide sequence coding for the signal peptide, the full-length caudex cDNA sequence analysis revealed that stenodactylin is encoded by a 1572-bp open reading frame (ORF) that encoded a polypeptide of 524 amino acids (Figure 1). Only the first 11 amino acids were obtained exclusively by Edman degradation.

The gene contains 753 bp that encode the A chain (251 amino acid residues with a calculated relative molecular mass (Mr) of 28,420.32) and 774 bp that encode the B chain (258 amino acid residues with a calculated Mr of 28,567.34) separated by a sequence of 45 bp that encodes the connecting peptide (Figure 1). The probable C-terminal end of the A chain and the connecting peptide were estimated based on the homology with volkensin.

Stenodactylin contains a total of 15 cysteine residues. The A chain includes Cys9, Cys157 and the C-terminal Cys246, which is involved in the intermolecular disulfide bond. The B chain includes 12 cysteines (Cys4, Cys20, Cys39, Cys59, Cys63, Cys78, Cys149, Cys162, Cys188, Cys191, Cys195, Cys206), eight of which (Cys20-Cys39, Cys63-Cys78, Cys149-Cys162, and Cys188-Cys206) form conserved intramolecular disulfide bridges; one cysteine (Cys4) at the N-terminal binds to the A chain. The amino acid residues that are important for the enzymatic activity of RIPs were conserved within the sequence of the A chain of stenodactylin (Tyr74, Tyr113, Glu163, Arg166, and Trp200).

In addition, based on the online program NetNGlyc1.0 [19], two possible glycosylation sites were detected at position Asn93-Gly94-Thr95 and Asn133-Val134-Thr135 in the B chain. This is noteworthy because, although N-glycosylation does not affect the catalytic activity of RIPs, it can affect their intracellular routing, their cytotoxicity and their immunogenicity [20,21].

**Figure 2:**
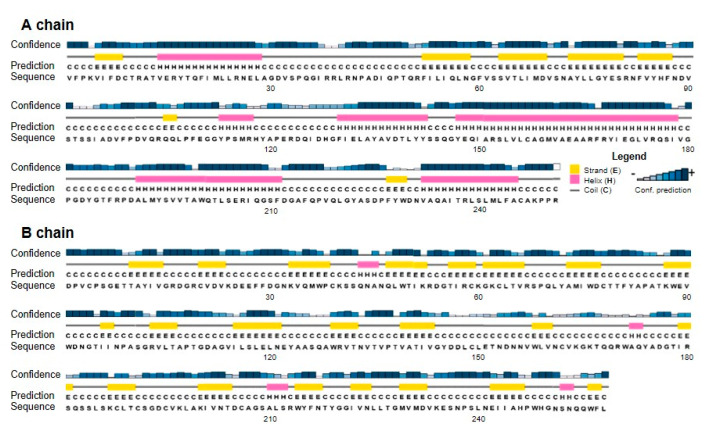
Secondary structure analysis of stenodactylin A and B chains. The secondary structure motifs were predicted using the PSIPRED Protein Structure Prediction Server. The predicted helix (H, pink) and strand (E, yellow) structure elements and randomly structured coil regions (C) of the target sequences are displayed according to the symbols shown in the legend. The confidence levels of the prediction are reported in the figure.

Based on the amino acid sequence, the secondary structure was predicted by the highly accurate PSIPRED algorithm for protein secondary structure prediction [22]. The 509 amino acid-long protein was calculated to have 25.7% extended strands, 21.6% α helices and 52.7% random coils, with α-helix structures mainly present in the A chain (Figure 2).

**Figure 3:**
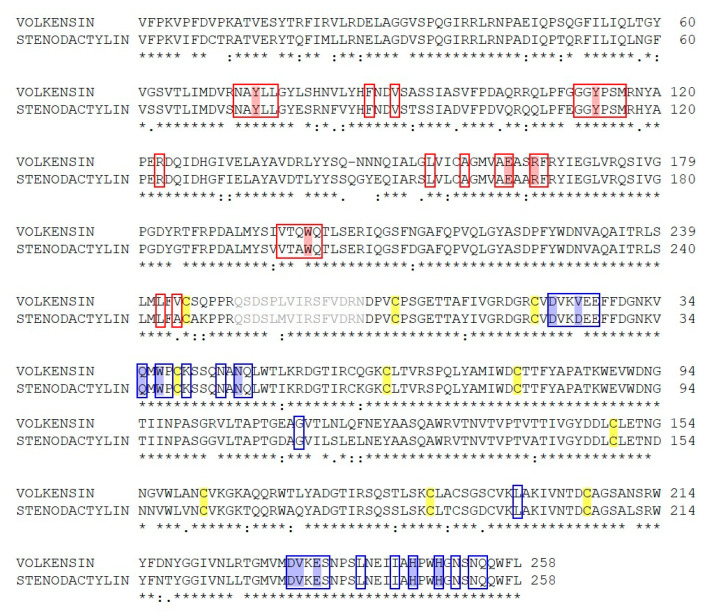
Alignment between stenodactylin and volkensin (GenBank CAD61022). Identical residues (*), conserved substitutions (:) and semiconserved substitutions (.) are reported. The A and B chains are presented in black; the sequence of the linker peptides is presented in gray. The putative amino acids that are present in the active site pocket (boxed in red) or in the galactoside-binding sites (boxed in blue), those involved in substrate binding or catalysis (highlighted in red), those involved in sugar binding (highlighted in blue), and those involved in disulfide bridges (highlighted in yellow) are represented, and they were assigned by comparison with the structure of ricin (accession no. 2AAI, 3RTI and 3RTJ). The dash indicates a gap introduced into the sequences to maximize alignments.

The stenodactylin polypeptide sequence was aligned with volkensin using the Clustal Omega software [23]. A comparison of the amino acid sequence of stenodactylin with volkensin showed 86.1% amino acid identity (Figure 3). This homology was not surprising because these two RIPs were purified from plants belonging to the same genus (*Adenia*). The results show higher identity between the B chains (90.3%) than between the A chains (81.7%). In addition, the A chain of stenodactylin contains one more cysteine at position 9 compared with volkensin. The B chains of both stenodactylin and volkensin contain 12 cysteine residues.

The catalytic key residues that are involved in the enzymatic mechanism and the 25 amino acids that are involved in the active center of the A chain (Figure 3) are almost conserved, except for Ala199 and Ala245, in stenodactylin, which are replaced with Gln198 and Val244, in volkensin.

### 2.2. Structure of Stenodactylin

To ascertain the main structural characteristics of stenodactylin, a three-dimensional structure was predicted by comparative modelling using several type 2 RIP crystal structures as templates. The selected best model was found to have a confidence score (C-score) of 0.64, template modelling (Tm) score of 0.80 ± 0.09, and root-mean-square deviation (RMSD) of 6.0 ± 3.7 Å, which satisfied the range of parameters for molecular modelling.

**Figure 4:**
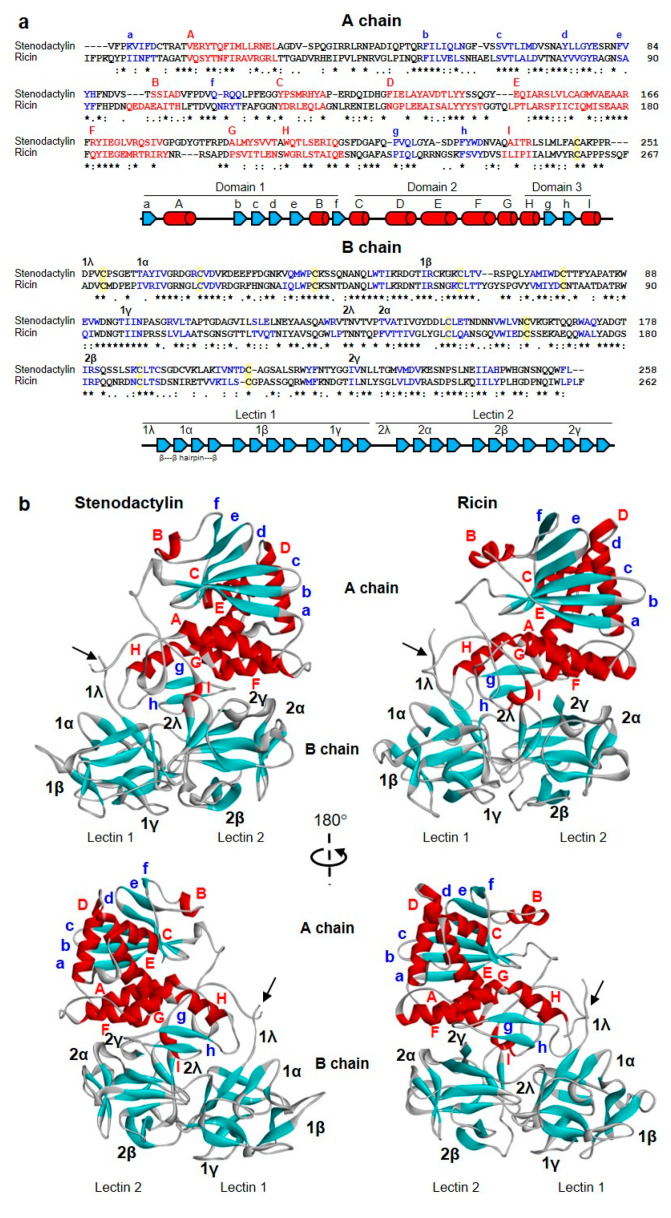
Structure of stenodactylin compared with ricin. (**a**) Amino acid sequence alignment of the A and B chains of stenodactylin and ricin. The β strands (blue), the α helices (red) and the cysteines involved in the disulfide bonds (highlighted in yellow) are indicated. The helices are labelled A to I and the strands of the β sheets are labelled a to h in the A chain. The domains and subdomains in the B chain are also indicated. Identical residues (*), conserved substitutions (:) and semiconserved substitutions (.) are reported. The cartoons represent the different structural motifs in both A and B chains. (**b**) Three-dimensional structure of stenodactylin compared with ricin (Protein Data Bank accession no. 2AAI). The three-dimensional structural modelling was carried out on the I-TASSER server and the figure was generated using Discovery Studio 2016. The α helices (red), the β chains (cyan), and the coils (grey) are represented. The helices are labelled A to I and the strands of the β sheets are labelled a to h in the A chain. The structural domains and subdomains in the B chain are also indicated. Arrows indicate the position of the disulfide bond linking A and B chains.

Even though stenodactylin shares a low amino acid sequence identity with both ricin and abrin-a (Table 1), it has a 3D structure similar to that of ricin [24] and abrin-a [25] (Figure 4, Figure S2). Stenodactylin is formed by a 251 amino acid A-chain bound to a B-chain of 258 amino acids by a disulfide bond in which Cys246 of the A chain and Cys4 of the B-chain participate.

The A chain can be divided into three folding domains that come together creating a deep active site pocket. This is common, not only to A-chains of type 2 RIPs but also to type 1 RIPs [26]. Domain 1 extends from the N-terminus to residue 109 and consists of six β-strands (strands a to f) and two α-helices (helices A and B) alternating in the order aAbcdeBf (Figure 4a). The six β-strands are arranged in a β-sheet of antiparallel strands sitting on domain 2 (Figure 4b). In domain 1, the Tyr74 that participates in the binding of adenine is located. Domain 2 is composed of residues from 110 to 199 and consists of five α-helices (C–G helices) containing the catalytic amino acids Glu163 and Arg166 and the other amino acid that binds adenine (Tyr113). Domain 3 extends from residue 200 to the C-terminus and consists of an α-helix-β-fork-α-helix (HghI) motif that is characteristic of A chains of type 2 RIPs and type 1 RIPs derived from type 2 RIPs by B-chain deletion. This structural motif has been related to the ability of these proteins to cross membranes [26] and contains the Trp200 that closes the active site.

**Figure 5:**
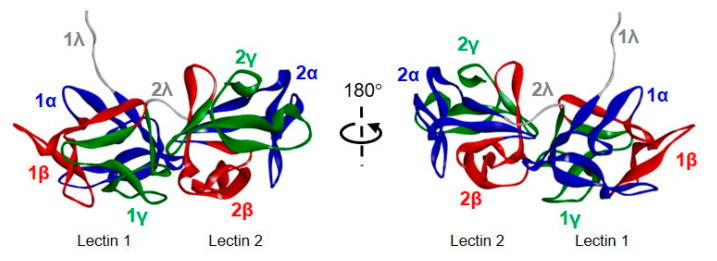
Structure of stenodactylin B chain. The three-dimensional structural modelling was carried out on the I-TASSER server and the figure was generated using Discovery Studio 2016. The structural domains and subdomains in the B chain are indicated.

Stenodactylin B-chain is composed of two homologous lectins, each of them consisting of four subdomains, λ, α, β and γ (Figure 4a). Subdomain 1λ (residues 1 to 9) participates in the disulfide bonding of the A- and B-chains and subdomain 2λ (residues 131 to 137) connects the two lectins of the B-chain. Lectin 1 contains the homologous subdomains 1α (residues 10 to 56), 1β (residues 57 to 94) and 1γ (residues 95 to 130), which are organized in a β trefoil fold. Each subdomain consists of a β-strand, a β-fork, and another β-strand. The β-strands form a six-strand β-barrel and the three β-forks form a lid on the barrel (Figure 4b, Figure 5). This structure is repeated in lectin 2 with subdomains 2α (residues 138 to 178), 2β (179 to 221) and 2γ (221 to 258). The central structure of the β-strands of lectins 1 and 2 is very similar in stenodactylin and ricin, while the main differences are found in the loops that stand out from these central structures (Figure 4b).

### 2.3. Sequence Comparison between Stenodactylin and Other RIPs

The sequence of stenodactylin was aligned and compared with the sequences of other RIPs, including both type 2 (toxic and non-toxic) and type 1.

The alignment and comparison are important (i) to identify both conserved and non-conserved amino acids, (ii) to understand the degree of evolutionary change and (iii) to detect amino acids that are important for their enzymatic activity.

**Figure 6:**
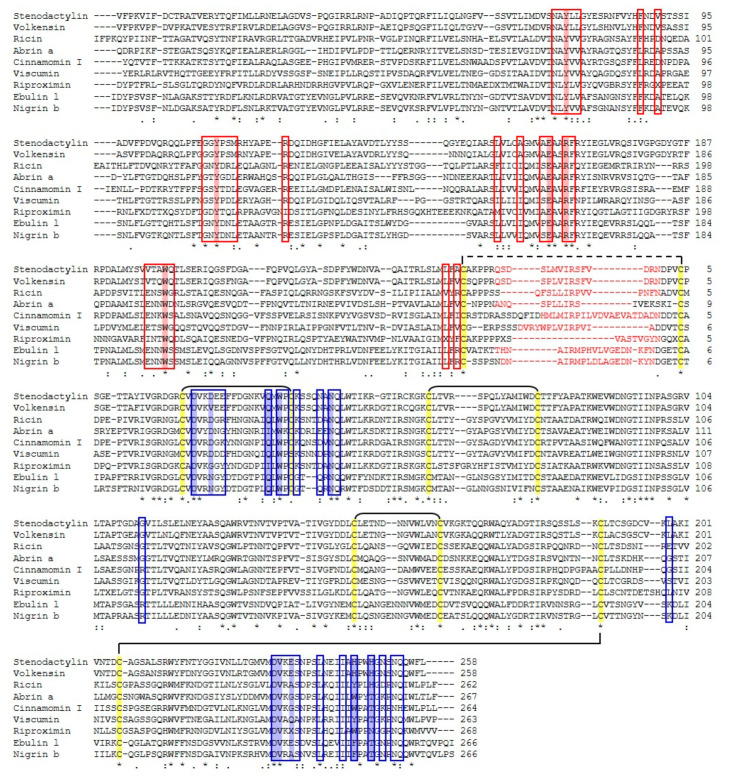
Protein sequence alignment of stenodactylin with volkensin (accession no. CAD61022), ricin (accession no. P02879), abrin a (accession no. P11140), cinnamomin I (accession no. AAF68978), viscumin (accession no. P81446), riproximin (accession no. CAJ38823), ebulin l (accession no. CAC33178), and nigrin b (accession no. P33183). Identical residues (*), conserved substitutions (:) and semiconserved substitutions (.) are reported. The A and B chains are presented in black and the sequence of the connecting peptide is presented in red. The putative amino acids that are present in the active site pocket (boxed in red) or in the galactoside-binding sites (boxed in blue), those involved in substrate binding or catalysis (highlighted in red), those involved in sugar binding (highlighted in blue), and those involved in disulphide bridges (highlighted in yellow) are represented, and they were assigned by comparison with the structure of ricin (accession no. 2AAI, 3RTI and 3RTJ). Dashes denote gaps introduced into the sequences to maximize alignments.

The amino acid sequence of stenodactylin was aligned with the amino acid sequences of toxic type 2 RIPs (e.g., volkensin, ricin, abrin, viscumin, riproximin) and non-toxic (e.g., cinnamomin, ebulin l and nigrin b) that have been reported in GenBank (Table 1, Figure 6). The multiple alignment analysis showed that, in all RIP evaluated, the B chains contain eight cysteine residues, involved in four conserved intramolecular disulfide bridges, and the B chain N-terminal cysteine that forms the intermolecular disulfide bridge between the A and B chains. Furthermore, the catalytic key residues (Tyr74, Tyr113, Glu163, Arg166, Trp200 in stenodactylin) that are involved in the enzymatic mechanism and the binding of adenine are conserved in all A chains of the RIPs.

Table 1 reports the identity between stenodactylin with toxic and non-toxic type 2 RIPs and type 1 RIPs. The results show that there is a high percentage of identity between the A and B chains of stenodactylin and volkensin. All other identities were lower, ranging from 27.7 to 33.0% for A chains and from 42.1 to 47.7% for B chains. The identity with type 1 RIPs is very low, being 18.1, 18.9 and 24.0% with dianthin, saporin and momordin, respectively. 

**Figure 7:**
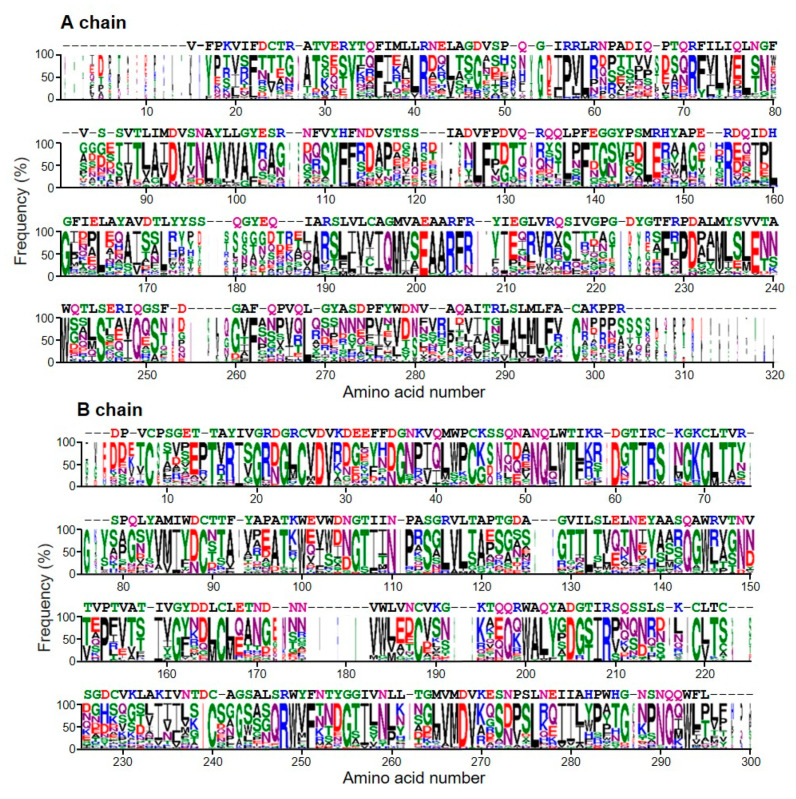
Sequence logos of the A and B chains of type 2 RIPs. The sequence logo representation of the alignment of the A and B-chain sequences from 46 representative type 2 RIPs belonging to 28 plant species was created as indicated in the “Materials and Methods” section. Letter height is proportional to the frequency of that amino acid at that position in the alignment respect to all the amino acids; letter width is proportional to the frequency of that amino acid but includes gaps. The sequence of stenodactylin is indicated above the logos.

The sequence of A- and B-chains of stenodactylin compared with the logos of type 2 RIPs from 28 plant species are shown in Figure 7. These logos are representative of all type 2 RIPs of the plant kingdom as each species is represented by one, two or three sequences. The A chain is the least conserved, but still has 24 almost invariable amino acids (with a frequency greater than 90%). Four of these amino acids are located in the active site (Tyr143, Glu201, Arg204 and Trp241 of the logo, which correspond to the residues Tyr113, Glu163, Arg166, and Trp200 of stenodactylin). It is worth mentioning that residue 98 in the active site is frequently (83%) Tyr (Tyr74 in stenodactylin), but it may be replaced by other amino acids. Ser245 (204 in stenodactylin) is not in the active site but is adjacent to and stabilizes the Trp of the active site. The other invariant amino acids are located outside and away from the active site and it has been postulated that they could participate in the internal dynamics of the enzyme [26]. Stenodactylin has two changes in these amino acids (Pro57 and Ala291 in the logo are Arg38 and Ser240 in stenodactylin, respectively).

The B chain is much more conserved (Figure 7) and has 52 amino acids with a frequency greater than 90%, and only changes Leu116 by Arg103 in stenodactylin. Some of these amino acids are located in the 1α (Asp27, Val28, Asn51, Gln52 that correspond to residues 22, 23, 46 and 47 of stenodactylin) and 2γ (Asp268, Val269, Leu276, Gln291 which correspond to residues 232, 233, 240 and 254 of stenodactylin) sites, but most of them are outside and even away from sugar binding sites. It has been suggested that these amino acids could complete the symmetry of both β trefoils or could constitute a structure that expands from the 1α site to the 2γ site participating in a network of protein motions that functionally connect both sugar binding sites [26]. Finally, it should be mentioned that only four amino acids directly involved in the binding of sugars (Asp27 and Asn51 in the 1α site, Asp268 and Val269 in the 2γ site) are highly conserved.

### 2.4. Phylogenetic Analysis

**Figure 8:**
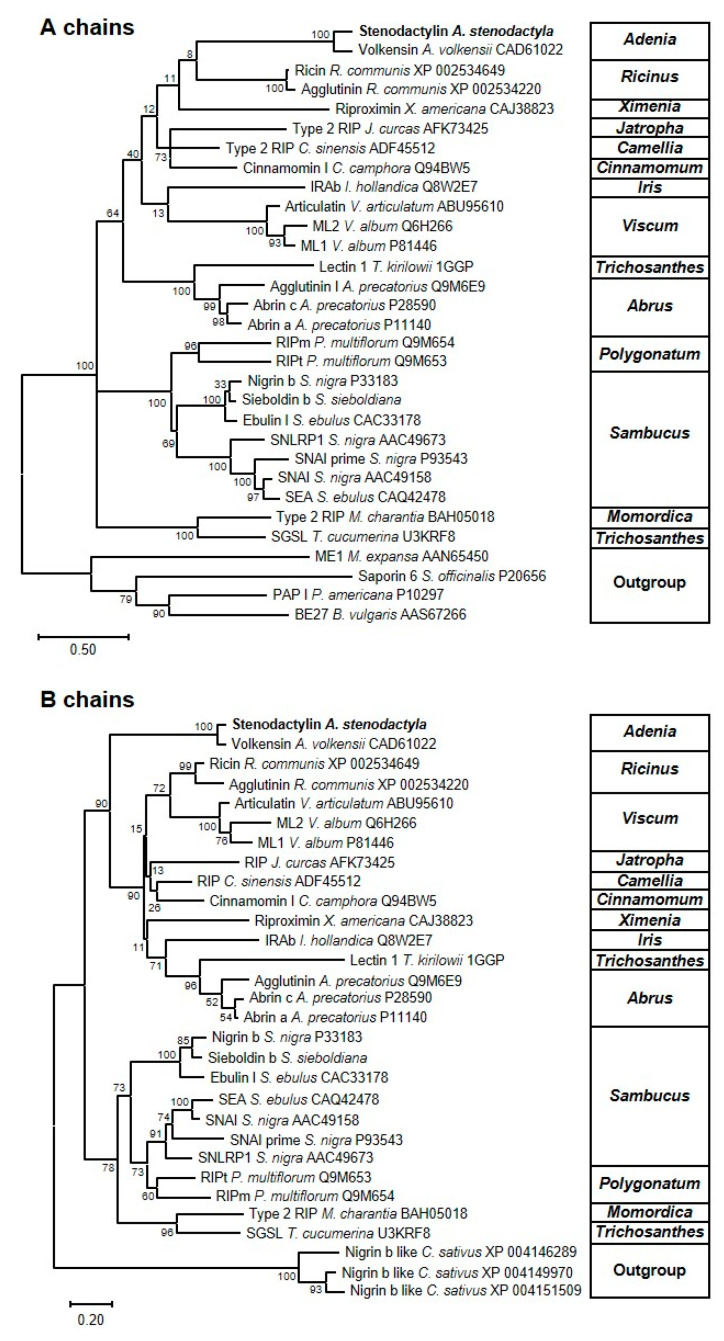
Molecular phylogenetic analysis by the Maximum Likelihood method of representative A and B-chain type 2 RIPs. The evolutionary history was inferred as indicated in the “Materials and Methods” section. The sequences of representative type 1 RIPs and monomeric lectins were used as the outgroup for the A and B chains, respectively. The name of the RIP (if any), the species and the accession number are indicated. All the sequences were retrieved and processed as indicated in the “Materials and Methods” section.

To understand the relationship between stenodactylin and other type 1 and type 2 RIPs (both toxic and non-toxic), phylogenetic trees were constructed based on the amino acid sequences of the A and the B chains of 11 families (Passifloraceae, Euphorbiaceae, Olacaceae, Theaceae, Lauraceae, Iridaceae, Santalaceae, Cucurbitaceae, Fabaceae, Asparagaceae and Adoxaceae) (Figure 8). The B-chain phylogenetic tree proved to be more reliable, with higher bootstrapping values for most of the branches. In this phylogenetic tree, proteins are segregated into two major clusters. One of them contains the non-toxic RIPs from the genera *Sambucus*, *Polygonatum*, *Momordica* and *Trichosanthes*; the other contains the toxic RIPs (including ricin and stenodactylin) but also non-toxic RIPs from the genera *Jatropha*, *Camellia*, *Cinnamomum*, *Iris* and *Trichosanthes*. Although the A-chain phylogenetic tree displays lower bootstrapping values (especially in the toxic RIP branch), it mostly reflects the B-chain tree. These phylogenetic trees suggest that toxic RIPs, such as stenodactylin or ricin, may appear in the evolution from a branch of non-toxic RIPs.

## 3. Discussion

In this paper, the complete amino acid sequence of stenodactylin was determined. Moreover, analysis of homology between stenodactylin and other RIPs was evaluated.

Sequence analysis showed that stenodactylin A and B chains contain 251 and 258 amino acids, respectively, corresponding to calculated Mr of about 28 kDa for both chains. The sugar presence could explain the difference between the molecular weight of the B chain based on the amino acid sequence (28 kDa) and the molecular weight observed by electrophoretic mobility (32 kDa) [7]. As reported for other type 2 RIPs, the A chain of stenodactylin contains only two lysines. Lysine residues are potential ubiquitination sites, and the small number of lysines is important to avoid ubiquitination and subsequent degradation [27]. In type 2 RIPs, the A and B chains are linked by a disulfide bridge between two cysteines. In ricin, the two cysteines that are involved are Cys259 of the A chain and Cys4 of the B chain [28]. Similarly, in stenodactylin Cys246 of A chain forms the disulfide bridge with Cys4 of B chain.

Based on the ricin A chain sequence, the key residues of active site are Tyr80, Tyr123, Glu177, Arg180 and Trp211 [15,29]. Near the active site, six other amino acids are important and conserved in both mono and bi-chain RIPs. These residues (Asn78, Arg134, Gln173, Ala178, Glu208 in ricin) are not directly involved in the depurination mechanism, but they help maintain the catalytic conformation [28,30]. The same amino acids of the active site are also conserved in the stenodactylin A chain (Tyr74, Tyr113, Glu163, Arg166, and Trp200).

As reported in the Introduction section, ricin B chain folds into two globular domains, each of which is composed of three subdomains. Only 1α and 2γ subdomains are involved in the galactose binding. The amino acids involved in the binding of galactose in the 1α site of ricin are Asp22, Asp25, Gln35 and Trp37, while the ones constituting the 2γ binding site are Asp234, Val235, Ala237, Tyr248 and His251 (Figure 6) [31]. Analysis of stenodactylin showed that all the amino acids that are involved in the first binding site of ricin are fully conserved (Asp22, Asp25, Gln35 and Trp37). In the 2γ binding site of stenodactylin, two amino acids are changed, compared to ricin; Ala237 and Tyr248 are replaced with Glu235 and His246 in stenodactylin, respectively. The substitution of Tyr by His was observed in volkensin [32], *R. communis* agglutinin [33] and *P. multiflorum* RIP [34]. Site-directed mutagenesis studies on the ricin B chain demonstrated that the replacement of Tyr248 with His248 reduced its binding activity [35]. The presence of a positive charge within the 2γ binding site prevents the hydrophobic interaction between the pyranose ring of galactose and the aromatic ring of Tyr; this substitution reduced functionality [34]. Cinnamomin contains three substitutions in the 2γ binding site compared with ricin (Gly239 for Ala237, Trp250 for Tyr248 and Thr253 for His251) (Figure 6). While the first substitution was conserved and the second one was between two aromatic amino acids, the third one removes a positive charge. As the lectin activity requires strictly conserved amino acids in both the 1α and 2γ domains, their change may explain the reduced cytotoxicity of cinnamonin [36]. Consistently, Tyr248 in ricin is replaced with Phe249 in ebulin l. These changes reduce the affinity of ebulin l for galactose-containing glycoproteins or glycolipids of the plasma membrane surface, thus reducing the cytotoxicity of the molecule [37,38]. On the other hand, non-toxic type 2 RIPs that are specific for sugars other than galactose have been reported. Thus, the tetrameric type 2 RIPs from species of the genus *Sambucus* (SNAI, SEA and SSA) are specific for Neu5Ac/galactose [39] and *Iris x hollandica* type 2 RIPs are specific for galactose/mannose [30]. In the case of the tetrameric RIPs from *Sambucus*, only the 2γ site is functional, because the 1α site has an additional cysteine involved in the linking between the two B-chains of the tetramer. Changes in the binding amino acids of the 2γ site, Glu235 by Gln, His246 by Tyr and His249 by Thr or Asn (Figure S3), allow the binding of galactose but also sialic acid [39]. In the type 2 RIPs from *Iris x hollandica* (IRAb and IRAr), changes occur in both 1α and 2γ sites, e.g., Trp37 by Ser and His246 by Trp (Figure S3), which allow the binding of both galactose and mannose [30].

All these data suggest that the 2γ binding site is important for the toxicity of RIPs, and any changes in this site could affect RIP binding. However, this hypothesis does not correlate with the results obtained with stenodactylin. In fact, stenodactylin, despite a change in this subdomain, is one of the most toxic RIPs.

According to the data reported in the literature [40], a high degree of identity was detected when the B chain of stenodactylin was compared with other B chains (90.3% with volkensin and ranging from 42.1 to 47.7% with the other RIPs). These data support the hypothesis that the B chain is a product of a gene duplication event [41]. The comparison of the stenodactylin A chain sequence with type 1 RIPs showed a very low level of identity. As above reported, the stenodactylin A chain showed a low degree of identity when compared with other type 2 RIP A chains, except for volkensin. Moreover, the identity of the A chains was lower than the identity of the B chains. The homology between the type 1 RIPs and the stenodactylin A chain was even lower than the identity calculated between the stenodactylin A chain and other type 2 RIP A chains. 

The high cytotoxic potential of stenodactylin together with its ability to elicit in cancer cells a rapid stress response, leading mainly to apoptosis, but also triggering other cell death pathways, makes stenodactylin an ideal candidate as a pharmacological molecule for drug targeting in the experimental treatment of several cancer diseases. Due to its high systemic toxicity, native stenodactylin could only be employed for loco-regional treatments in cancer therapy. However, stenodactylin A-chain linked to a specific carrier by chemical conjugation or by genetic engineering, could find application to specifically target tumor cells in systemic therapy [11]. Moreover, stenodactylin may have an application in neurobiology. In fact, its characteristic retrograde transport in the peripheral nerves and central nervous system and its ability to kill neurons through different death pathways could be exploited to develop new molecular tools for experimental models of neurodegenerative diseases [9,10].

The knowledge of the stenodactylin sequence and structure may help to identify the amino acids directly or indirectly involved in RIP toxicity, thus stimulating research about this protein.

## 4. Materials and Methods 

### 4.1. Materials

Stenodactylin was purified from the caudex of *Adenia stenodactyla* as described by Stripe and co-workers [7]. Adenia plants were purchased from Exotica Botanical Rarities (Erkelenz-golkrath, Germany) and, if not used immediately on arrival, were kept in the greenhouse of the Botanical Garden of the University of Bologna.

For RT-PCR analysis, the GenElute Mammalian Total RNA Kit and the primers were purchased from Sigma-Aldrich (St. Louis, MO, USA). For stenodactylin sequence the total RNA was isolated using the RNeasy Minikit, whereas the plasmids were purified by QIAfilter plasmid purification kit, both purchased from Qiagen (Hilden, Germany). The PCR products were purified using the High Pure PCR Product Purification kit obtained from Roche Applied Science (Penzberg, Germany). The reverse transcriptase MuLV, the dNTPs were obtained from GeneAmp RNA PCR kit (Roche). The chemically competent *E. coli* INVαF’ and the pCR^®^II cloning vectors were purchased from Invitrogen (Carlsbad, CA, USA).

The iScript cDNA synthesis Kit and the SsoFast™ EvaGreen^®^ Supermix were obtained from Bio-Rad (Hercules, CA, USA). Other reagents used were from Merck (Darmstadt, Germany), Carlo Erba (Milano, Italy) and from Sigma.

Protein concentration was determined by UVICON 860 Spectrophotometer (Kontron Instruments, Milano, Italy). The DNA content was determined by a Beckman DU 640 spectrophotometer (Beckman, Brea, CA, USA). RT-PCR was performed using the CFX96 Real-Time PCR System (Bio-Rad). 

PCR was conducted using the thermal cycler PCR system 2400 (Perkin Elmer, Waltham, MA, USA). Using DNA sequencing software, a search for sequence similarity was performed with the BLAST program available online [42]. The multiple sequence alignment program Clustal Omega [23] was used to detect the extent of sequence conservation and the secondary structure prediction was carried out using PSIPRED [22]. Glycosylation sites were predicted using the NetNGlyc1.0 server [19].

### 4.2. Methods

#### 4.2.1. Synthesis of cDNA

The caudex of *A. stenodactyla* was disrupted using mortar and pestle and grinded to a fine powder under liquid nitrogen. Approximately 100 mg of total RNA was isolated using the RNeasy Minikit (Qiagen) according to the manufacturer’s instruction. Poly(A)-rich RNA was reverse transcribed using the synthetic oligonucleotide T1 (5′ CGTCTAGAGTCGACTAGTGC(T)20 3′). Approximately 2 µg of total RNA and 1 µL of RNAse inhibitor were incubated at 65 °C in a thermal cycler for 5 min. It was later cooled on ice for 1 min and 15 µL of reaction mixture containing: 1 × PCR Buffer II, 5 mM of MgCl_2_, 1 mM of each dNTP, 10 µM of T1, and 2.5 U/µL of MuLV reverse transcriptase (RNA PCR kit. Roche) was added. The reaction mixture was incubated for 20 min at 23 °C, then for 20 min at 42 °C, and finally 5 min at 99 °C.

The specific primers for the stenodactylin gene sequence were designed and synthesized based on the volkensin sequences (Table 2). Three pairs of primers were used to amplify the full-length cDNA sequence of stenodactylin: STA2-STB1R for A-chain; STA2-STB3R for A-chain and a piece of B-chain; STB1-STB5R for B-chain.

For cDNA amplification, 2 µL of the above-synthesize cDNA was used and 16 µL of master mix and 0.5 µM of each primer were added. A typical reaction master mix included: 1 × PCR buffer/Mg^2+^, 0.25 mM dNTPs Mix, 2.5 U Taq Polymerase (Biotools, Madrid, Spain). PCR amplification was performed with the following conditions: an initial denaturation at 94 °C for 5 min, followed by 35 cycles of 94 °C for 30 s, 55–54 °C for 45 s and 72 °C for 2 min, and an additional extension of 10 min at 72 °C.

About 5 μL of amplified products from each tube was analyzed on 0.8% agarose gel.

As reported in Figure S1, the amplicons of expected size (about 0.85 kb for the A and B chains alone and about 1.1 kb for the combined A and small segment of B chains) were obtained, and the PCR products were purified using the High Pure PCR product Purification Kit (Roche) according to the manufacturer’s instruction.

#### 4.2.2. cDNA Cloning and Sequence

The three purified PCR fragments were ligated into the pCR^®^II vector and then were used to transform the Chemically Competent *E. coli* INVαF’ (Invitrogen) according to the manufacturer’s instructions. Two clones for each fragment were purified and sequenced using M13 primers. DNA sequencing was carried out on the CENIT Support system (Villamayor-Salamnca, Spain).

#### 4.2.3. Sequence Retrieval and Data Treatment

All the amino acid sequences of ribosome-inactivating proteins and lectins used in this study are available in the National Center for Biotechnology Information (NCBI) sequence database (https://www.ncbi.nlm.nih.gov/protein/), except those from sieboldin and SSA from *Sambucus sieboldiana* (Miq.) Blume ex Graebn., which were obtained from [43,44], respectively. For the representation logo and phylogenetic analysis, the signal peptide, and connecting peptide were removed using the following criteria by order of preference: information in the data bank entry, information in the literature from N-terminal sequencing, comparison with other close related sequences, and the use of the SignalP-5.0 Server (http://www.cbs.dtu.dk/services/SignalP/) [45].

#### 4.2.4. Secondary Structure Prediction

The secondary structure was predicted using the PSIPRED Protein Structure Prediction Server (http://bioinf.cs.ucl.ac.uk/psipred/) [22].

#### 4.2.5. Sequence Alignment

Sequence alignment was performed using the ClustalW tool included in the Mega X suite (https://www.megasoftware.net/) [23] with default parameters and edited manually to align the amino acids Tyr, Tyr, Glu, Arg, Trp in the active site of the A-chains, and all the Cys in the B-chains. Then, the sequences included between each pair of conserved amino acids were aligned automatically, and finally the complete sequences as well. Multiple sequence alignments were graphically represented by sequence logos [46] created with WebLogo 3 (http://weblogo.threeplusone.com/) [47]. The logos were created by using the A and B-chain sequences from 46 representative type 2 RIPs belonging to 28 plant species and limited to three, the maximum number of sequences for each species. For the representation of Figure 3, Figure 4 and Figure 6, the alignment was carried out using the Clustal Omega server (https://www.ebi.ac.uk/Tools/msa/clustalo/) [48].

#### 4.2.6. Protein Structure Studies and Graphical Representation

The structure of ricin (accession number 2AAI) is available in the Protein Data Bank (https://www.rcsb.org/). Three-dimensional structural modelling of stenodactylin was carried out on the I-TASSER server (https://zhanglab.ccmb.med.umich.edu/I-TASSER/) [49]. Study and graph representations of protein structures were performed with the aid of the Discovery Studio Visualizer suite (v16.1.0) (https://www.3dsbiovia.com/).

#### 4.2.7. Phylogenetic Analysis

The evolutionary histories of the A and B chains were inferred by using the Maximum Likelihood method and either the Whelan and Goldman [50] (for the A chain) or the JTT matrix-based [51] (for the B chain) models. The trees with the highest log likelihood (−13,115.61 for the A chain and −10,193.09 for the B chain) are shown. The percentage of trees in which the associated taxa clustered together is shown next to the branches. Initial trees for the heuristic search were obtained automatically by applying Neighbor-Join and BioNJ algorithms to a matrix of pairwise distances estimated using the JTT model, and then selecting the topology with superior log likelihood value. A discrete Gamma distribution was used to model evolutionary rate differences among sites (two categories (+G, parameter = 1.9285 for the A chain and 1.1766 for the B chain)). In the case of the A chain, the rate variation model allowed for some sites to be evolutionarily invariable (+I, 1.01% sites). The trees are drawn to scale, with branch lengths measured in the number of substitutions per site. This analysis involved 31 and 30 amino acid sequences for the A and B chains, respectively. There was a total of 346 and 314 positions in the final dataset for the A and B chains, respectively. Evolutionary analyses were conducted in MEGA X [23].

## Figures and Tables

**Table 1:** Identity of eight type 2 RIPs and three type 1 RIPs with stenodactylin.

	RIP Name	Identity (%) Stenodactylin A Chain	Identity (%) Stenodactylin B Chain	Identity (%) Stenodactylin Whole Molecule
Type 2	Volkensin	81.7	90.3	86.1
	Ricin	31.4	47.7	40.3
	Viscumin	31.0	46.0	38.1
	Abrin a	31.2	44.6	37.9
	Riproximin	27.7	43.3	35.8
	Cinnamomin	33.0	42.1	37.3
	Ebulin l	28.7	44.2	36.5
	Nigrin b	29.0	43.9	36.5
Type 1	Saporin	18.9		
	Dianthin	18.1		
	Momordin	24.0		

**Table 2:** Primer sequence.

Primer	Sequence
STA2	5′ GCCACGGTAGAGAGRTACACT 3′
STB1R	5′ AAGTCGTCTCCCCGGAAGGGC 3′
STB3R	5′ GGCGGGGTTGATGGTTCC 3′
STB1	5′ TGCCCTTCCGGGGAGACGACT 3′
STB5R	5′ TAGGAACCATTGCTGGTTGGA 3′

## Supplementary Materials

The following are available online at https://www.mdpi.com/2072-6651/12/9/538/s1, Figure S1: Isolation of stenodactylin amplicons, Figure S2: Superimposition of stenodactylin and ricin structures, Figure S3: Alignment between the sugar-binding subdomains of stenodactylin, volkensin, ricin, abrin-a, SNAI, SSA, SEA, IRAb and IRAr.
