# Supplementary material for: Primary Sequence and 3D Structure Prediction of the Plant Toxin Stenodactylin
Source: Toxins (Basel). 2020 Aug 21;12(9):538. doi: 10.3390/toxins12090538 (PMC7551084; doi:10.3390/toxins12090538)
Supplement: Supplementary file 1 [file toxins-12-00538-s001.pdf]

Supplementary Materials: Primary Sequence and 3D Structure Prediction of the Plant Toxin Stenodactylin

Rosario Iglesias, Letizia Polito, Massimo Bortolotti, Manuela Pedrazzi, Lucía Citores, José M. Ferreras and Andrea Bolognesi

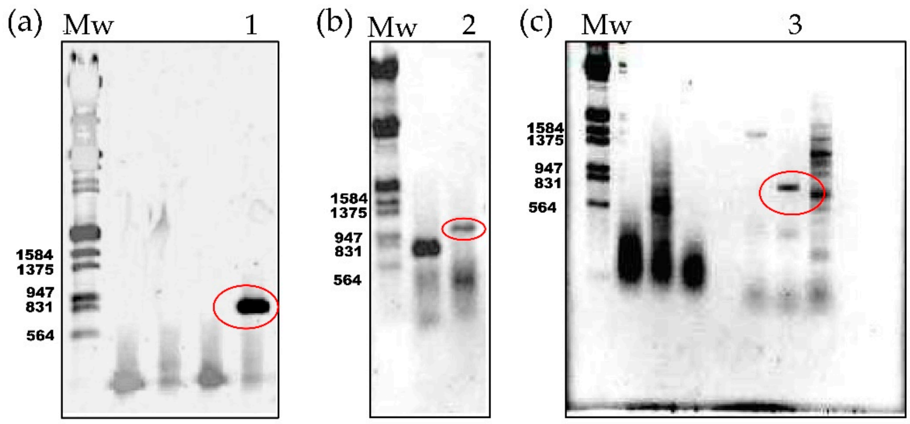

Figure S1. Amplification of stenodactylin A-chain (**a**, lane 1), A-chain and a part of B-chain (**b**, lane 2), B-chain (**c**, lane 3). MW: λ Hind III/EcoRI double digest DNA marker. The red circles indicate the amplificate.

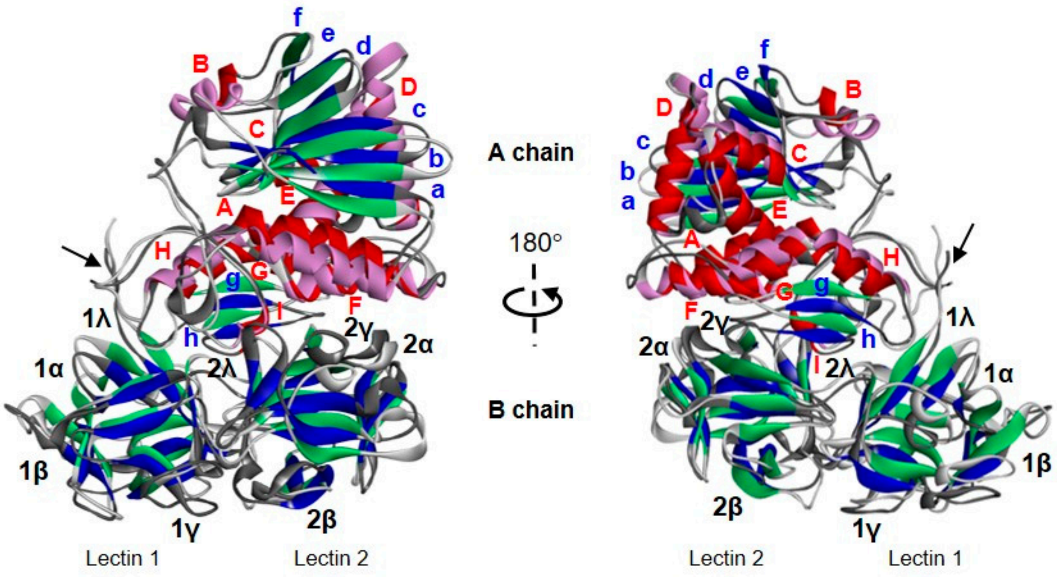

Figure S2. Superimposition of stenodactylin and ricin structure (Protein Data Bank accession number 2AAI). The three-dimensional structural modelling was carried out on the I-TASSER server and the figure was generated with Discovery Studio 2016. The α helices (red and purple in stenodactylin and ricin, respectively), the β chains (blue and green in stenodactylin and ricin, respectively), and the coils (dark and light grey in stenodactylin and ricin, respectively) are represented. The helices are labelled A to I and the strands of the β sheets are labelled a to h in the A chain. The structural domains and subdomains in the B chain are also indicated. Arrows indicates the position of the disulfide bond linking A and B chains.

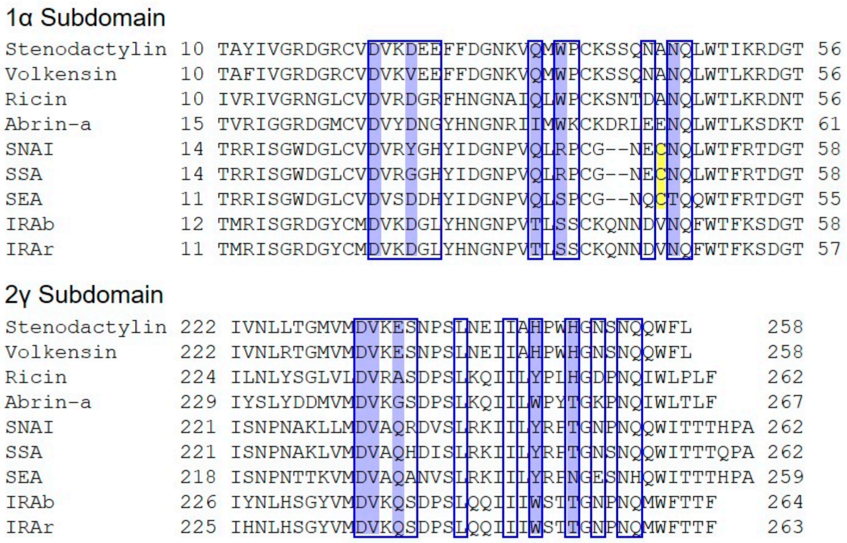

Figure S3. Alignment between the sugar-binding subdomains of stenodactylin, volkensin (acc.no CAD61022) ricin (acc.no P02879), abrin-a (acc.no P11140), SNAI (acc.no AAC49158), SSA, SEA (acc.no CAQ42478), IRAb (acc.no Q8W2E7) and IRAr (acc.no AAL55093). The putative amino acids that are present in the sugar-binding sites (boxed in blue) and those involved in sugar binding (high-lighted in blue) are represented, and they were assigned by comparison with the structure of ricin (acc.no 2AAI, 3RTI and 3RTJ). The cysteines involved in disulfide bridges between B-chains of tetrameric RIPs are high-lighted in yellow. The dash indicates a gap introduced into the sequences to maximize alignments.
